# Supplementary material for: Expressive dynamics models with nonlinear injective readouts enable reliable recovery of latent features from neural activity
Source: ArXiv. 2023 Sep 12:arXiv:2309.06402v1. Preprint. [Version 1] (PMC10516113)
Supplement: 1 [file NIHPP2309.06402V1-supplement-1.pdf]

Expressive dynamics models with nonlinear injective readouts enable reliable recovery of latent features from neural activity
Supplementary Material

G Datasets

G.1 Simulated neural data

G.1.1 Latent trajectories

We used the Arneodo system [42] to generate synthetic data because it exhibits mildly chaotic behavior (Lyapunov exponent equal to 0.243), it has a low-dimensional state space, and the regions around its fixed points are well-sampled by trajectories of the system. As demonstrated by [15], these properties allow recovery of latent dynamics in the absence of a nonlinear embedding. The Arneodo system is described by the following system of equations

$$\dot{x} = y \tag{13}$$
$$\dot{y} = z \tag{14}$$
$$\dot{z} = -ax - by - cz + dx^3 \tag{15}$$

where $a = -5.5$, $b = 4.5$, $c = 1.0$, and $d = -1.0$ [42].

The system was simulated using the `dysts` Python package, which offered well-reasoned standards for initial conditions, integration steps, and resampling frequency [47]. Initial conditions had been determined by running the model until the moments of the autocorrelation function were stationary. Integration steps had been chosen based on the highest significant frequency observed in the power spectrum. After integration, trajectories were resampled to contain 35 points per period, where period was based on the dominant frequency in the power spectrum.

G.1.2 Embedding low-dimensional trajectories on a nonlinear manifold

We simulated neural activity by nonlinearly embedding the Arneodo trajectories as firing rates in the neural space. First, the trajectories were linearly projected into the neural space via a set of encoding vectors γ_i and standardized for each neuron (see Methods). These activations $\mathbf{a}_i$ were passed through a sigmoid with input scaling η_i and output scaling $b = 2$ to produce reasonable firing rates as follows:

$$\eta_{\mathbf{i}} = 10^{0.8 \cdot \frac{i-1}{N-1} + 0.2}, \tag{16}$$
$$\mathbf{y}_i = \psi_i(\mathbf{a}_i) = b \times \sigma(\eta_i \times \mathbf{a}_i), \quad i = 1, 2, \ldots, N. \tag{17}$$

where $\sigma(\cdot)$ denotes the sigmoid function. This resulted in a set of activation functions $\psi_i(\cdot)$ ranging from quasi-linear to step-like. The resulting rates $\mathbf{y}_i$ were used to parameterize a Poisson process, which was sampled to obtain spiking data for N neurons ($N = 12$).

G.1.3 Embedding low-dimensional trajectories onto linear manifold

For Figure 2A, we tested whether Linear-NODEs fit to linearly-embedded data would find non-injective readouts when $\hat{D} > D$. We simulated an alternative dataset with the same procedure as above, except instead of passing the activations $\mathbf{a}_i$ through the sigmoidal non-linearity, we exponentiated them to find the rate parameter $\mathbf{y}_i$ of a Poisson process, which was sampled to obtain spiking data for N neurons ($N = 12$). These data were used only in Figure 2A.

G.2 Real neural data

The maze dataset was previously collected from the motor cortex of a monkey performing a reaching task [38]. This dataset has been widely used to characterize the dynamics of motor cortical activity [38, 27, 44]. In particular, these data are well-modeled by autonomous dynamics [44].

The monkey was trained to perform a delayed reaching task in which it had to maintain its hand at the center of a 2D maze displayed on a screen while a target was shown somewhere within the maze. After a randomly-timed delay, a go-cue was issued which prompted the monkey to move its hand from the center of the screen to the indicated target. Each trial also had a set of obstacles (i.e., the walls of a maze) with various configurations that required the monkey to produce reaches with varied trajectories, even when they were directed towards the same target. A total of 108 of these maze configurations (i.e., target and obstacle combinations) are included in this dataset.

Neural activity was recorded using two Utah arrays [48], one in the dorsal premotor (PMd) cortex and one in the primary motor cortex (M1) [38]. Threshold crossings were sorted offline. The dataset contained 182 neurons in total, of which 137 were included in the held-in set and the remaining 45 were part of the held-out set. The held-out neurons were used to calculate the co-smoothing bits-per-spike metric (K.2.1). The monkey's hand and cursor positions were recorded during the experiment (K.2.2).

These data were downloaded from the Distributed Archives for Neurophysiology Data Integration (DANDI, [49]). We binned spike counts at 20 ms and trialized and aligned the data to 250 ms before and 450 ms after movement onset. Further details can be found in [38, 27].

H Model training

H.1 Simulated neural data

All weights were initialized from $\mathcal{U}(-\sqrt{k}, \sqrt{k})$, where $k = 1/\text{in\_features}$ for linear layers and $k = 1/\text{hidden\_size}$ for the GRU encoder weights. Dropout layers ($p = 0.05$) were inserted before and after the initial condition linear projection during training. We used the average Poisson negative log-likelihood (NLL) across neurons and time points as our training objective. Models were trained incrementally to improve the stability of training: rather than compute loss on the whole trajectory, we added groups of 5 new time steps every 75 epochs, up to the max of 70 steps. Models were trained by stochastic gradient descent using Adam for 3000 epochs. A single learning rate was shared for the optimizer of the encoder, generator, and readout weights for each model. Each generator was a NODE that contained an MLP with six hidden layers, each with 128 ReLU units.

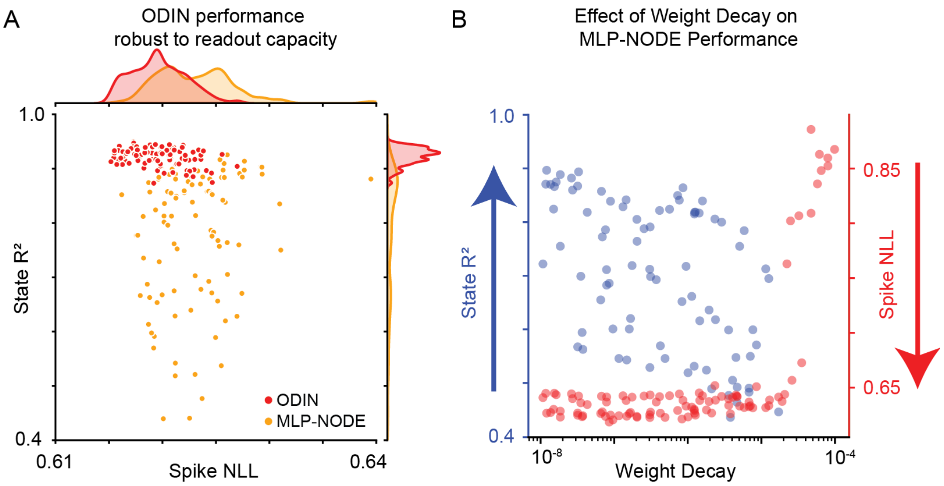

Figure S1: Example hyperparameter sweeps for ODIN and MLP-NODE

We performed initial hyperparameter sweeps to determine ranges that resulted in good reconstruction performance as measured by Spike NLL (see Methods), and used the same hyperparameter setting for models across state dimensionalities. Two example hyperparameter sweeps testing the effect of readout capacity (100 model initializations with readout hidden sizes in [60,200] and number of hidden layers in [1,3]) and weight decay (100 model initializations with weight decay drawn log-uniformly from [1e-8, 1e-4], Supp. Fig. S1). We found that across readout capacities, good reconstruction performance implied good latent recovery for ODIN but not MLP-NODE. Additionally, we found that increasing weight decay on MLP-NODE tended to degrade rather than improve latent recovery. Across all HPs tested, we found no hyperparameter settings for which ODIN had good reconstruction performance but poor latent recovery.

HPs for models trained on the Arneodo system are given in Table S1.

Table S1: Training hyperparameters (Synthetic Data)

	Arneodo		
	Linear	MLP	ODIN
Batch Size	650	650	650
Learning Rate	2e-3	1.88e-4	1.88e-4
Encoder Hidden Size	100	100	100
Dropout	0.05	0.05	0.05
NODE Hidden Layers	6	6	6
NODE Hidden Size	128	128	128
Readout Hidden Layers	0	2	2
Readout Hidden Size	-	150	150

H.2 Real neural data

The weight initialization procedure and dropout settings were the same as for the models trained on Arneodo. In addition to Poisson NLL, we also added regularization terms (L_2 norm on weights) and used different learning rates for the encoder, generator, and readout modules. We trained these models using Adam for 1500 epochs with the loss function given by Equation 18:

$$L(\mathbf{x}, \hat{\mathbf{y}}, \theta_E, \theta_G, \theta_R) = \text{PoissonNLL}(\mathbf{x}|\hat{\mathbf{y}}) + \lambda_E||\theta_E||_2^2 + \lambda_G||\theta_G||_2^2 + \lambda_R||\theta_R||_2^2 \tag{18}$$

where $\mathbf{x}$ and $\hat{\mathbf{y}}$ represent the observed spiking activity and the predicted firing rates, respectively, and $\lambda_E, \lambda_G, \lambda_R$ represent the regularization coefficients for the L_2 regularization penalty applied to the model weights $\theta_E, \theta_G, \theta_R$ of the encoder, generator, and readout, respectively. To improve training stability, we also used different learning rates for each component of the model ($\alpha_E, \alpha_G, \alpha_R$). Specific parameters for models trained on the Maze dataset are given in Table S2.

H.2.1 AutoLFADS

We trained AutoLFADS models of varying latent dimensionalities as a point of reference for ODIN's performance [50]. Notably, we used the autonomous version of LFADS and fixed the initial condition, generator, and factors dimensionality to $\hat{D}$ for these experiments. The batch size was 512 and the encoder hidden size was 100. Population-Based Training was used with a population of 20 workers to search initial learning rate (init: 1e-2, range: loguniform; 1e-5, 5e-2), dropout rate (init: 5e-2, range: uniform; 0.0, 0.6), coordinated dropout rate (init: 0.3, range: uniform; 0.01, 0.7), initial condition KL (range: loguniform; 1e-10, 1e-3), generator L2 scale (range: loguniform; 1e-10, 1e0), and encoder L2 scale (range: loguniform; 1e-10, 1e0). Linear ramp-up of KL and L2 penalties occurred over the first 80 epochs and the population was subjected to binary tournament and perturbation every 25 epochs for a total of 1000 training epochs.

I Injectivity estimation

To demonstrate the approximate injectivity of the Flow readout, we tested whether the readout could be inverted to recover the inferred latent activity. The readout mapping $\hat{g}$ should satisfy the following

Table S2: Training hyperparameters (Maze Data)

	Maze	
	Linear	ODIN
Batch Size	64	64
λ_E	1.6e-5	2.2e-6
λ_G	1.6e-5	1.35e-9
λ_R	1.6e-5	4.2e-6
α_E	5e-3	4e-4
α_G	5e-3	7e-4
α_R	5e-3	1.4e-4
Encoder Hidden Size	100	100
Dropout	0.05	0.05
NODE Hidden Layers	6	6
NODE Hidden Size	128	128
Readout Hidden Layers	0	3
Readout Hidden Size	-	128
Number of Flow Steps	-	25

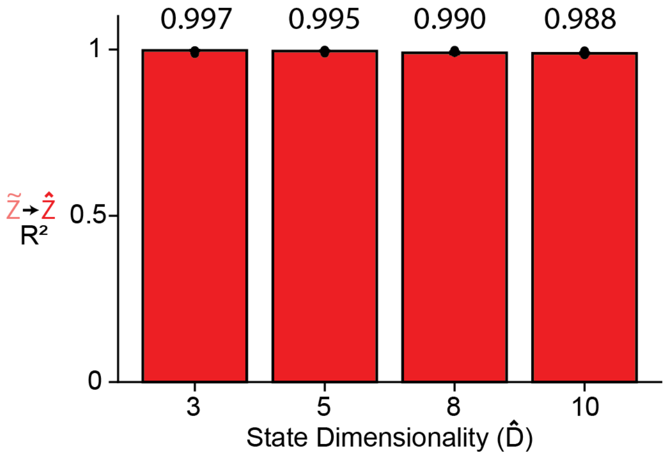

Figure S2: Injectivity of the Flow readout across state dimensionalities. Each bar indicates the mean value of 5 randomly initialized ODIN models for each state dimensionality. Results from individual models are plotted as points.

equations

$$\tilde{\mathbf{z}}_t = \hat{g}^{-1}(\hat{g}(\hat{\mathbf{z}}_t)) \tag{19}$$

$$\tilde{\mathbf{z}}_t \approx \hat{\mathbf{z}}_t \tag{20}$$

where $\hat{\mathbf{z}}_t$ is the inferred latent activity and $\tilde{\mathbf{z}}_t$ is the latent activity recovered by the reverse pass of the Flow.

We computed the R^2 between the inferred and recovered $\mathbf{z}_t$ for these models and found that our mappings were able to recover the inferred $\hat{\mathbf{z}}_t$ with average R^2 values across randomly initialized models of 0.997, 0.996, 0.990, and 0.988 at $\hat{D} = 3, 5, 8, 10$, respectively (Supplementary Figure S2).

I.1 Effective Rank

To assess the injectivity of the Linear readout, we used a previously published method that determines the approximate number of significant singular values of a given matrix A [41]. Let A be a complex-valued, non-all-zero matrix of size $N \times \hat{D}$, where $N > \hat{D}$ that acts as the weight matrix of a readout from inferred latents $\hat{\mathbf{z}}$ to predicted log-rates $\log \hat{\mathbf{y}}$ in the equation $\log \hat{\mathbf{y}} = A\hat{\mathbf{z}} + \mathbf{b}$. We perform a singular value decomposition (SVD) on A, such that $A = U\Delta V$, where U and V are unitary matrices

of size $N \times N$ and $\hat{D} \times \hat{D}$, respectively, and Δ is an $N \times \hat{D}$ rectangular diagonal matrix containing the real non-negative singular values $\sigma_1 \geq \sigma_2 \geq \ldots \geq \sigma_{\hat{D}} \geq 0$.

For simplicity, let us define $\sigma = (\sigma_1, \sigma_2, \ldots, \sigma_{\hat{D}})^T$. We then compute the singular value distribution p_k, for $k = 1, 2, \ldots, \hat{D}$, as

$$p_k = \frac{\sigma_k}{\|\sigma\|_1}, \tag{21}$$

where $\|\sigma\|_1$ is the L_1-norm. Using this singular value distribution, we can calculate the Shannon entropy H as

$$H(p_1, p_2, \ldots, p_{\hat{D}}) = -\sum_{k=1}^{\hat{D}} p_k \log(p_k). \tag{22}$$

The authors in [41] define the effective rank of the matrix A, denoted as $\text{erank}(A)$, using the Shannon entropy H as follows:

$$\text{erank}(A) = \exp\left(H(p_1, p_2, \ldots, p_{\hat{D}})\right). \tag{23}$$

The effective rank gives us a measure of the number of significant singular values in A. As traditional rank counts a matrix as being "full-rank" even if it has negligibly small but non-zero singular values, the effective rank provides a more informative assessment of the matrix's rank when used as the readout from a NPDM. We assessed the effective rank of the linear readout for 5 Linear-NODE models (with state dimensionality of $\hat{D} = 2, 3, 5, 8, 10$, respectively) trained on synthetic neural data generated by linearly embedding trajectories from the Arneodo system (Section G.1.1) into log-firing rates, and found that while the reconstruction performance improved as $\hat{D}$ increased, the effective rank plateaued at erank ≈ 4 (Fig 2A, Supp. Fig. S3).

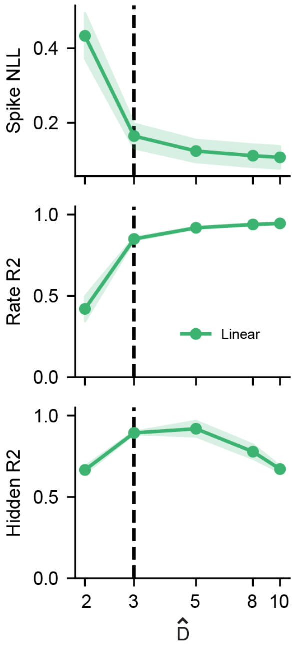

Figure S3: Linear-NODE trained on synthetic neural activity from linearly-embedded Arneodo system

I.2 Cycle Consistency

To directly compare injectivity of the Flow readout versus the MLP, we quantified how well each model's inferred latent activity could be recovered from the reconstructed log-rates. To do this, we took our fully-trained 10D ODIN and MLP-NODE models (shown in Fig. 2C, $\hat{D} = 10$) and obtained the inferred latent activity $\hat{\mathbf{z}}$ and predicted log-firing rates $\log \hat{\mathbf{y}}$ from the Arneodo dataset. Then, we trained a separate network $h : \log \mathbf{Y} \to \mathbf{Z}$ to minimize the mean squared error between its output $\tilde{\mathbf{z}}$ and the model-inferred latent activity $\hat{\mathbf{z}}$ (see Table S3 for hyperparameters).

$$\tilde{\mathbf{z}} = h\left((\log \hat{\mathbf{y}})\right) \tag{24}$$

We computed the coefficient of determination between the re-generated latent activity $\tilde{\mathbf{z}}$ and inferred latent activity $\hat{\mathbf{z}}$. If this performance is high, the inferred latents can be recovered from the log-rates suggesting that the readout is approximately injective.

$$\text{Cycle Consistency} = R^2(\tilde{\mathbf{z}}, \hat{\mathbf{z}}) \tag{25}$$

Table S3: Training hyperparameters (Cycle-Consistency MLP, h)

Parameter	Value
Batch Size	2048
Learning Rate	1e-3
Hidden Layers	3
Hidden Size	128
Epochs	1000

It is possible for a readout to be fully injective (i.e., that $\hat{g}^{-1}$ exists), but still compress some features of latent activity into negligibly small contributions to the predicted firing rates, making the readout effectively, if not technically, non-injective. We reasoned that if this were the case, the inverse mapping h, in order to properly invert the warping applied by $\hat{g}$, would be highly sensitive to noise. We expect that such noise perturbations would be warped by h into large changes in the predicted latents. Using the models trained without noise, we computed the R^2 of re-generated latents $\tilde{\mathbf{z}}$ compared to the inferred latents $\hat{\mathbf{z}}$. We therefore consider both the noise-free and noise-corrupted cycle consistency scores as indicators of the approximate injectivity of each readout, taking into consideration undue distortion applied in the process of learning the injective mapping.

$$\tilde{\mathbf{z}}_\sigma = h(\log \hat{\mathbf{y}} + \epsilon_\sigma), \quad \epsilon_\sigma = \mathcal{N}(0,\, \sigma), \;\; \sigma \in [0.01, 0.05, 0.1, 0.2, 0.5]) \tag{26}$$

$$ccR^2_\sigma = R^2(\tilde{\mathbf{z}}_\sigma, \hat{\mathbf{z}}) \tag{27}$$

I.3 Alternative injective readout

As an additional confirmation that injectivity was the critical addition to non-linear readouts that made latent recovery more robust, we tested an alternative injective architecture — an invertible neural network (INN) [20]. We found that using a 6-layer INN in place of the Flow readout had comparable Rate R^2 and State R^2 to ODIN, and that, like ODIN, State R^2 was stable as $\hat{D}$ increased beyond $D = 3$ (see Supp. Fig. S4). Each INN layer was composed of coupling, permutation and affine transformations. Additional training parameters are noted in S4. This result further supports our claims that injective networks empirically promote robust latent recovery.

Unfortunately, the INN hidden layer size is obligated to be the size of either the input or output dimensionalities, whichever is larger. Therefore, in realistic biological datasets where the number of neurons can be highly variable across datasets, the capacity of the INN readout is intrinsically linked to the number of recorded neurons. For this reason, we chose to use the Flow readout, which decouples the computational capacity of the injective transformation from the dimensionality of the neural space.

Table S4: Training hyperparameters INN (Synthetic Arneodo Data)

Parameter	Value
Batch Size	650
Learning Rate	1.88e-4
Encoder Hidden Size	100
Dropout	0.05
NODE Hidden Layers	6
NODE Hidden Size	128
Readout Hidden Layers	6
Readout Hidden Size	12

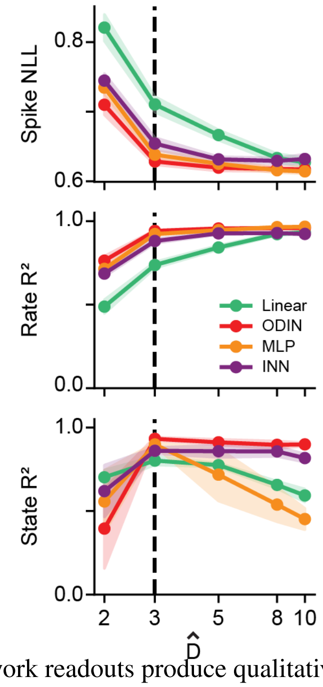

Figure S4: Invertible Neural Network readouts produce qualitatively similar results to Flow readout models. Data shown is the same as Fig. 2C, except overlaid with INN readout model (purple)

J Fixed point finding and characterization

For each model (Linear-NODE, MLP-NODE and ODIN), we located fixed points (FPs) by finding the positions in the latent space that minimized the norm of the vector field via the objective $q = \frac{1}{2}||\hat{f}||_2^2$ [1, 40]. We initialized our search with 1024 randomly sampled initial states from along inferred latent trajectories. We used Adam with a learning rate of 5e-2 to minimize the q-value for each point independently over 10,000 iterations. Candidate points that did not achieve a q-value less than a magnitude of 7e-3 were excluded. As more than one candidate can approach the same FP, we combined candidate points that were within a specified distance, ϵ = 1, from one another. In practice, points that were excluded had much larger q-values than the putative fixed points. We then linearized the dynamics around each FP and computed the system Jacobian to determine the stability and rotational character of the system around these FPs.

K Metrics

K.1 Synthetic data metrics

K.1.1 Rate reconstruction (Rate R^2)

We computed the coefficient of determination between true ($\mathbf{Y}$) and predicted ($\hat{\mathbf{Y}}$) rates for each neuron, and reported the average value across neurons.

$$\text{Rate } R^2 = R^2(\mathbf{Y}, \hat{\mathbf{Y}}) = \frac{1}{N}\sum_{i=0}^{N} 1 - \frac{\sum(\mathbf{y}_i - \hat{\mathbf{y}}_i)^2}{\sum(\mathbf{y}_i - \bar{\mathbf{y}}_i)^2}$$

K.1.2 Latent state reconstruction (State R^2)

To compute State R^2, we concatenated a vector of ones with the true latent states ($\mathbf{Z_1}$), then used the pseudoinverse to find the optimal affine transformation from the true latents to the inferred latents ($\hat{\mathbf{Z}}$) (i.e., optimal linear estimation). We computed the coefficient of determination (R^2) between the true and inferred latent activity with the same equation as in K.1.1.

$$\mathbf{W}_z = \mathbf{Z}_1^{\dagger}\hat{\mathbf{Z}} \tag{28}$$

$$\text{State } R^2 = R^2(\hat{\mathbf{Z}}, \mathbf{Z}_1\mathbf{W}_z) \tag{29}$$

K.1.3 Activation function comparison

We developed a method for deriving an estimate of the inferred activation functions $\hat{\psi}_i(\cdot)$ for a comparison to the true activation functions $\psi_{\mathbf{i}}(\cdot)$ (see Equation 17). We projected the true encoding vectors $\boldsymbol{\gamma}_i$ into the latent space of the model via the affine transformation $\mathbf{W}_z$ (see section K.1.2). We then used these encoding vectors $\hat{\boldsymbol{\gamma}}_i \in \mathbb{R}^{\hat{D}}$ to convert inferred latent states $\hat{\mathbf{Z}} \in \mathbb{R}^{T\times\hat{D}}$ into an activation $\hat{\mathbf{a}}_i \in \mathbb{R}^T$ for each neuron.

$$\hat{\gamma}_i = \boldsymbol{\gamma}_{1,i}\mathbf{W}_z, \text{ for } i = 1, 2, \cdots, N \tag{30}$$

$$\hat{\mathbf{a}}_i = \hat{\mathbf{Z}} \cdot \hat{\gamma}_i \tag{31}$$

To estimate the activation function for a given neuron i, we need pairs of inferred activations $\hat{\mathbf{a}}_i$ and firing rates $\hat{\mathbf{y}}_i$. For each neuron, we split firing rates into 20 quantiles and computed the corresponding median activation $\hat{\mathbf{a}}_{i,1:20}^{med}$ and firing rate $\hat{\mathbf{y}}_{i,1:20}^{med}$ within each quantile.

$$\hat{\mathbf{y}}_{i,1:20}^{med}, \hat{\mathbf{a}}_{i,1:20}^{med} = \text{Quantize}(\hat{\mathbf{y}}_i, \hat{\mathbf{a}}_i, 20) \tag{32}$$

We represented the inferred activation function $\hat{\psi}_i(\cdot)$ using these activation-firing rate pairs. We then performed the same procedure on the true rates and activations to find a similar representation of the true activation function $\psi_i(\cdot)$ for each neuron. To compare the true activation function $\psi(\cdot)$ to the estimated activation function $\hat{\psi}(\cdot)$, we combined the activations of each neuron i and its corresponding firing rate as the columns of the matrices:

$$\hat{\boldsymbol{\Psi}}_i = \begin{pmatrix}\hat{\mathbf{a}}_i^{med} & \hat{\mathbf{y}}_i^{med}\end{pmatrix}, \quad \boldsymbol{\Psi}_i = \begin{pmatrix}\mathbf{a}_i^{med} & \mathbf{y}_i^{med}\end{pmatrix}$$

Because the inferred latent activity can be scaled and translated arbitrarily with respect to the true latent activity, we found the optimal affine transformation between $\hat{\boldsymbol{\Psi}}_i$ and $\boldsymbol{\Psi}_i$. We used the R^2 of this mapping to quantify the correspondence between the two activation functions $\hat{\psi}_i(\cdot)$ and $\psi_i(\cdot)$ for each neuron.

K.2 Neural Latents Benchmark metrics

K.2.1 Co-smoothing bits-per-spike (co-bps)

A common failure mode of many dynamics models is to find latent activity that can accurately reconstruct the firing rates of neurons seen by the encoders, but fails to reconstruct neural activity of

held-out neurons. To avoid this pitfall, we used a previously developed metric called co-smoothing bits-per-spike which evaluates reconstruction performance on a set of held-out neurons not visible to the encoders [27]. At a high-level, this metric quantifies how well the firing rates of the held-out neurons can be predicted from the spiking of the held-in neurons (see G.2). This metric is defined by Equation 33 for each held-out neuron.

$$\text{co-bps} = \frac{1}{n_s \log 2}(\mathcal{L}(\hat{\mathbf{y}}_{n,t};\mathbf{x}_{n,t}) - \mathcal{L}(\bar{\mathbf{y}}_{n,:};\mathbf{x}_{n,t})) \tag{33}$$

where $\bar{\mathbf{y}}_{n,:}$ is the mean firing rate for neuron n across time, n_s is the total number of spikes for that neuron, $\hat{\mathbf{y}}_{n,t}$ is the predicted firing rate from the model at time t, $\mathbf{x}_{n,t}$ represents the observed spiking of that neuron at time t, and $\mathcal{L}$ represents the Poisson log-likelihood. More information can be found in [27].

K.2.2 Velocity decoding $\mathbf{R}^2$

A common metric of performance is how well inferred firing rates can be used to predict behavioral variables, as this can be used downstream for decoding intent in clinical applications like brain-computer interfaces [51]. For the Maze dataset, hand velocity has been shown to be highly correlated with the neural firing in motor cortices. We compute this metric using the method from [27], in which a ridge regression model is trained to predict the observed hand velocity from inferred firing rates. The coefficient of determination (R^2) was then evaluated on validation data that was not used to train the ridge regression velocity decoder.

L Compute resources

We used an internal computing cluster with a total of 30 Nvidia GeForce RTX 2080 Ti GPUs for model training. Each model trained on simulated neural data took approximately 3 hours to train, while each model trained on real biological data took approximately 1.5 hours to train. With 2 models training on each GPU, the 100 models included in Figs. 2, 3, 4, and 5 took approximately 150 GPU-hours and the 50 NODE-based models included in Fig. 6 took approximately 37.5 GPU-hours. FP finding was fast, requiring 1 minute for each model.

M Open-source packages used

- `torch` [52] (BSD license): Deep learning framework providing layer definitions, GPU acceleration, automatic differentiation, optimization, and more.
- `pytorch_lightning` (Apache 2.0 license): Lightweight wrappers for model training.
- `ray.tune` [53] (Apache 2.0 license): Distributed hyperparameter tuning.
- `dysts` [47] (Apache 2.0 license): Implementations for modeled dynamical systems.
- `fixed_point_finder` [40] (Apache 2.0 license): Inspiration for `torch`-based fixed point finder.
- `FrEIA` (MIT license): Implementation of alternative Invertible Neural Network architecture.
- `scikit-learn`[54] (BSD License): Implementations of linear regression models and principal component analysis.
